# Retrovirus-Mediated Expression of E2A-PBX1 Blocks Lymphoid Fate but Permits Retention of Myeloid Potential in Early Hematopoietic Progenitors

**DOI:** 10.1371/journal.pone.0130495

**Published:** 2015-06-22

**Authors:** Mark W. Woodcroft, Kyster Nanan, Patrick Thompson, Kathrin Tyryshkin, Steven P. Smith, Robert K. Slany, David P. LeBrun

**Affiliations:** 1 Department of Pathology and Molecular Medicine, Queen's University, Kingston, Ontario, Canada; 2 Department of Biomedical and Molecular Sciences, Queen's University, Kingston, Ontario, Canada; 3 Department of Genetics, University Erlangen, Erlangen, Germany; Emory University, UNITED STATES

## Abstract

The oncogenic transcription factor E2A-PBX1 is expressed consequent to chromosomal translocation 1;19 and is an important oncogenic driver in cases of pre-B-cell acute lymphoblastic leukemia (ALL). Elucidating the mechanism by which E2A-PBX1 induces lymphoid leukemia would be expedited by the availability of a tractable experimental model in which enforced expression of E2A-PBX1 in hematopoietic progenitors induces pre-B-cell ALL. However, hematopoietic reconstitution of irradiated mice with bone marrow infected with E2A-PBX1-expressing retroviruses consistently gives rise to myeloid, not lymphoid, leukemia. Here, we elucidate the hematopoietic consequences of forced E2A-PBX1 expression in primary murine hematopoietic progenitors. We show that introducing E2A-PBX1 into multipotent progenitors permits the retention of myeloid potential but imposes a dense barrier to lymphoid development prior to the common lymphoid progenitor stage, thus helping to explain the eventual development of myeloid, and not lymphoid, leukemia in transplanted mice. Our findings also indicate that E2A-PBX1 enforces the aberrant, persistent expression of some genes that would normally have been down-regulated in the subsequent course of hematopoietic maturation. We show that enforced expression of one such gene, *Hoxa9*, a proto-oncogene associated with myeloid leukemia, partially reproduces the phenotype produced by E2A-PBX1 itself. Existing evidence suggests that the 1;19 translocation event takes place in committed B-lymphoid progenitors. However, we find that retrovirus-enforced expression of E2A-PBX1 in committed pro-B-cells results in cell cycle arrest and apoptosis. Our findings indicate that the neoplastic phenotype induced by E2A-PBX1 is determined by the developmental stage of the cell into which the oncoprotein is introduced.

## Introduction

Acute lymphoblastic leukemia (ALL) is the most common pediatric cancer. Approximately 5% of ALL cases are associated with a reciprocal translocation between chromosome bands 1q23 and 19p13.3 [1]. In the vast majority of cases, this leads to the fusion of the *E2A* (also called *TCF3*) and *PBX1* genes, resulting at the protein level in fusion of the N-terminal two thirds of E2A with most of PBX1 to create the oncogenic transcription factor E2A-PBX1. Relative to other B-progenitor ALL subtypes, leukemic blasts with t(1;19) typically manifest a more mature immunophenotype characterized by expression of cytoplasmic μ heavy chain thus justifying the term “pre-B-cell ALL” to denote such cases [1;2].

Although t(1;19) is associated almost exclusively with pre-B-cell ALL, B-lymphoid disease is generally not replicated in murine models of E2A-PBX1 oncogenesis. In the initial transgenic mouse model, E2A-PBX1 expression directed by the Eμ enhancer resulted in T-progenitor lymphoma after a brief latency period [3]. More recently, Bijl *et al*. were successful in forcing the development of B-progenitor ALL by crossing CD3ε^-/-^ mice, which fail to develop mature T-cells, with transgenic mice expressing E2A-PBX1 under the control of lymphoid specific promoter/enhancer elements [4]. Some of these progeny developed leukemia that manifested B-lymphoid immunophenotypic features and immunoglobulin gene rearrangements after a long latency period, although in half of the cases a combined lymphoid/myeloid phenotype was evident. Therefore, these transgenic approaches have not addressed the need for an experimentally tractable model of E2A-PBX1-driven pre-B-cell leukemia in order to elucidate mechanistic aspects of E2A-PBX1-mediated oncogenesis in the context of B-lymphopoiesis.

Adoptive transfer of bone marrow cells that have been transduced *ex vivo* with an E2A-PBX1-expressing retroviral vector generally leads to an aggressive myeloproliferative neoplasm, although instances of T-progenitor ALL have also been observed [5;6]. These transplantation studies have not adequately delineated the impact of E2A-PBX1 on lymphopoiesis, in part because the phenotypic analyses performed on engrafted, E2A-PBX1-expressing cells were carried out after the recipient animals began to show signs of illness, when the bone marrow was densely packed with immature myeloid progenitors rendering it difficult to assess the effect of E2A-PBX1 on non-myeloid lineages. Furthermore, these experiments were carried out using retroviral vectors that did not allow transduced cells and their progeny to be distinguished on a cell-by-cell basis by flow cytometry.

Therefore, the need persists to investigate the hematopoietic impact of E2A-PBX1 on the fate of early progenitors in order to elucidate E2A-PBX1 function and inform the eventual development of an experimentally tractable model of E2A-PBX1-induced B-lymphoid ALL. In the present study, we establish stable, retrovirus-mediated expression of E2A-PBX1 in uncommitted hematopoietic progenitors or committed B-lymphoid progenitors and determine the hematopoietic and transcriptional consequences *in vivo* and *in vitro*.

## Materials and Methods

### Mice

BALB/c mice 8–12 weeks old were purchased from Charles River and housed in the Queen’s University Animal Care Facility. These animals were handled according to a protocol (LeBrun-2009-021) specifically approved for this study by the Queen's University Animal Care Committee. After transplantation, recipient mice were maintained on acidified water in micro-isolator units under sterile conditions.

### Cell lines and culture conditions

The cell lines 293T (ATCC CRL-3216) and NIH 3T3 were cultured in Dulbecco’s Modified Eagle Medium (DMEM) supplemented with 10% fetal bovine serum (FBS; PAA Laboratories Inc., Etobicoke, ON), 1× antibiotic/antimycotic solution (Gibco, Carlsbad, CA), and 2 mM L-glutamine. The OP9 cell line [7], a gift from Dr. Juan Carlos Zúñiga-Pflücker, Sunnybrook Health Sciences Centre, Toronto, Ontario, was cultured in Iscoves Modified Dulbecco’s Medium (IMDM) supplemented with 20% FBS (Hyclone, Logan, UT), 1× antibiotic/antimycotic solution, 10 μM β-mercaptoethanol and 2 mM L-glutamine. OP9 cells were subjected to 15 Gy of γ radiation from a ^137^Cs source prior to co-culture with fetal liver-derived hematopoietic progenitor cells.

### Plasmids and generation of retroviral supernatants

The MIEV-EP1b and MIEV-EP1b-L20A retroviral backbone plasmids were constructed by excising the complementary DNA for E2A-PBX1b or its L20A mutant, respectively, from pBlueScript-SK(-) with the restriction enzymes *Not*I and *Sal*I and ligating them into a MIEV retroviral backbone plasmid that had been prepared by digestion with the same enzymes [5]. For construction of MIEV-EP1b-N51S, the mutation-containing portion of E2A-PBX1 was excised from pGD-EP1b-N51S (a gift from Dr. Mark Kamps) with *Bsp*MI and *Nco*I and ligated into the MIEV-EP1b vector that had been digested with the same enzymes. All constructs were verified by DNA sequencing. Retroviral supernatants were generated by co-transfecting 293T cells with 7.5 μg of MIEV-based backbone plasmid and 7.5 μg MCV-ecopac retroviral packaging plasmid using standard calcium phosphate methodology. Supernatants were stored at -80°C.

### Retroviral transduction and bone marrow transplantation assays

Bone marrow was harvested from the femurs and tibias of 8 to 10 week-old female BALB/c mice. Lineage-depleted (lin^-^) cells were obtained using a lineage depletion kit (Miltenyi Biotec Inc., Auburn, CA) according to the manufacturer’s instructions. Following depletion, the cells were resuspended in pre-stimulation mix (IMDM containing 15% fetal bovine serum, 2 mM L-Glutamine, 1× antibiotic/antimycotic solution, 50 μg/mL gentamicin (Sigma Aldrich, Oakville, ON), 10 ng/mL interleukin-3 (IL-3), 10 ng/mL IL-6 and 50 ng/mL murine stem cell factor (SCF)); all recombinant cytokines were purchased from Peprotech. Twenty-four hours after the initial pre-stimulation period, 2.0 × 10^6^ cells were pelleted and resuspended in 2.7 mL of retroviral supernatant, 0.3 mL of IMDM containing 100 ng/mL IL-3, 100 ng/mL IL-6, 500 ng/mL SCF and 50 ug/mL polybrene was added and the cell suspensions were transferred to 6-well dishes. Cells were transduced by spinoculation at 1800*g* for 2 h at 25°C. Following the spin, retroviral supernatant was removed and replaced with fresh pre-stimulation mix. The next day, cells were subjected to a second round of transduction and then 4.0 × 10^5^ transduced cells were mixed with 2.0x10^5^ whole bone marrow cells in Hank’s balanced salt solution (HBSS) and injected into the tail vein of lethally irradiated (9 Gy) BALB/c recipient animals. Mice were sacrificed by cervical dislocation three weeks post-transplantation prior to displaying signs of morbidity. More generally, mice were handled according to protocol LeBrun-2013-022-Or approved by the University Animal Care Committee of Queen's University.

### Isolation, transduction and culture of primary hematopoietic progenitor cells

Fetal livers were isolated from E12.5–14.5 BALB/c embryos and homogenized in phosphate-buffered saline (PBS) containing 1 mM EDTA. Lin^-^ fetal liver progenitor (FLP) cells were purified as described above, re-suspended in retroviral supernatant containing 5 μg/mL polybrene and plated in a 6-well plate containing 7.0 × 10^4^ irradiated OP9 cells. The plates were spun for 2 h at 1800*g* at 25°C and then the retroviral supernatant was aspirated and replaced with 3 mL of OP9 media supplemented with 20 ng/mL of recombinant interleukin-7 (IL-7) or a 1:100 dilution of J558-IL-7-conditioned supernatant. For some experiments, cultures were additionally supplemented with 20 ng/mL SCF. Cells were transferred to fresh, irradiated OP9 feeder cells every 3–4 days in a manner that accorded with published guidelines for maintaining optimal OP9 cell density [8]. In order to establish expression of estrogen-regulatable E2A-PBX1 in B-lymphoid progenitors, "pre-B-1" cells were flow sorted from the bone marrow of adult mice and expanded *ex vivo* in the presence of IL-7 and OP9 cells, according to a published protocol [8]. These cells were then transduced with p210^BCR-ABL^ by co-culture with 293T packaging cells that had been transiently transfected with the retroviral backbone plasmid MIEVpuro BCR-ABL (a gift from Dr. Peter Greer, Queen's University) and MCV-ecopac plasmids and then selected in 1 μg/mL puromycin. After confirmation of IL-7- and OP9-independent growth and a B-lymphoid (CD19^+^, CD11b^-^) immunophenotype, these cells were infected by spinoculation with MSCVneo-EPΔ623ER, a gift of Dr. Mark Kamps, University of California San Diego, selected in 1 mg/mL G418 and cloned by limiting dilution in 96-well plates. Expression of EPΔ623ER in the clone designated 3PER12 was confirmed by immunoblotting.

### Flow cytometry

For immunophenotyping, cells were washed in PBS and re-suspended in PEB (PBS containing 2 mM EDTA and 0.5% bovine serum albumin (BSA)) at 1.0 × 10^7^ cells/mL. Anti-CD16/32 was added to block non-specific antibody binding to Fc receptors and then cells were incubated for 5 minutes on ice. Subsequently, 1.0 × 10^6^ cells were incubated on ice for 20 minutes in 100 μL of PEB containing antibodies to the surface markers of interest; antibody details are provided in S1 Table. Cells were washed twice with ice-cold PBS and re-suspended in 500 μL of PBS prior to analysis. Apoptosis assays were performed using the Annexin-V-PE apoptosis kit according to the manufacturer's instructions (EMD Millipore, Darrnstadt, DE). Cell cycle analyses were performed using DyeCycle Green according to the manufacturer's instructions (Life Technologies, Carlsbad, CA). Flow cytometry was performed using a FC500 cytometer (Beckman Coulter, Mississauga, ON). All data analysis was carried out using the FlowJo software platform (Treestar Inc., Ashland, OR).

### RNA extraction and quantitative RT-PCR

Total RNA was extracted using the RNeasy Midi kit according to the manufacturer's instructions (Qiagen, Valencia, CA) and 50 μg was used for each reaction. Quantitative reverse transcriptase polymerase chain reaction (qRT-PCR) for Bmi1 was carried out on an Mastercycler ep realplex^4^ instrument (Eppendorf Inc., Hauppauge, NY) using the TaqMan RNA-to-Ct One Step kit according to the manufacturer's instructions (Applied Biosystems, Carlsbad, CA) with the following TaqMan gene expression assays: *Bmi1* (Mm03053308_g1) and *B2m* (Mm00437762_m1). qRT-PCR for other transcripts made use of the Biorad iScript One-step RT-PCR kit and the following TaqMan assays: *Meis1* (Mm00487664_m1), *Mycn* (Mm00476449_m1), *Gata3* (Mm00484683_m1), *Myc* (Mm00487804_m1), *Rora* (Mm01173766_m1), *Il6* (Mm00446190_m1), *Kit* (Mm00445212_m1), *Pax5* (Mm00435501_m1), *Ebf1* (Mm00432948_m1), *B2m* (Mm00437762_m1) and *Tbp* (Mm00446971_m1). Relative transcript abundance was calculated using the standard ΔΔC_T_ method; *B2m* was used as the reference for *Bmi1* quantification and *Tbp* was used for the other transcripts. P-values shown or referred to in the figures were calculated using an unpaired, two-tailed Student's *t*-test.

### Immunoblotting

Immunoblotting was carried out as previously described using anti-E2A (sc-416; Santa Cruz Biotechnology, Santa Cruz, CA), anti-Ebf1 (Epitomics, Burlingame, CA; catalog number 3197–1), anti-Pax5 (Santa Cruz; catalog number sc-1974), anti-Bmi1 (EMD Millipore, Billerica, MA; catalog number 05–637) [9]. Anti-enhanced green fluorescent protein (GFP) (Roche, Laval, QC; catalog number 11814460001) or anti-**γ**-tubulin (Sigma; catalog number T5326) were used as loading controls.

### Chromatin immunoprecipitation

Chromatin immunoprecipitation (ChIP) followed by quantitative PCR (ChIP-qPCR) was carried out using 2–4 × 10^6^ cell equivalents of chromatin per immunoprecipitation (IP) according to a published protocol [10]. Immunoprecipitations were carried out using 2 μg of anti-H3K4me3 (EMD Millipore; catalog number 07–473) or anti-H3K27me3 (EMD Millipore; catalog number 07–449). Quantitative PCR was carried out using SYBR Green chemistry with primers (Ebf1-1, -2 and -3, and Pax5-2 and -3) that amplify regions within the *Ebf1* or *Pax5* promoters demonstrated previously to be differentially associated in B-lymphoid progenitors with the H3K27me3 versus H3K4me3 chromatin marks [11].

### Limiting dilution analysis

Serial dilutions corresponding to 1.5, 3, 6, 12, 24, and 48 cells per 100 μL were prepared in medium supplemented with IL-7 and SCF supernatants. For each dilution, 100 μl was plated in each of 16 wells of a 96-well plate; each well also contained 7.0 × 10^3^ irradiated OP9 cells. After 7 days, wells containing at least 50 viable cells were considered as indicating culture initiation and enumerated by microscopy. Data were analyzed using the extreme limiting dilution analysis (ELDA) tool [12]. Statistical significance was assessed using the χ^2^ test.

### Gene expression microarray analysis

E2A-PBX1-transduced FLPs and empty vector-transduced, FLP-derived, CD19^+^ pro-B-cells were maintained for approximately 40 days in the presence of IL-7 and SCF prior to purification of total RNA; the pro-B-cells were co-cultured with OP9 stromal cells. Total RNA was extracted as described above and quality was assessed using an Agilent Bioanalyzer (Agilent Technologies Canada Inc., Mississauga, ON). For each sample, 300 ng of RNA was used as a template for amplification and Cy3 labeling using the Agilent QuickAmp kit; labeling and amplification efficiency were assessed using a Nanodrop spectrophotometer. For each sample, 1.65 μg of labeled copy RNA was hybridized to a mouse whole genome 4 × 44 K gene expression array (Agilent). After 17 hours of hybridization, arrays were washed and scanned using an Agilent Microarray Scanner. Feature extraction software (10.5.1.1) was used to extract expression data from array images and to perform initial data preprocessing, including background correction. The data were then further preprocessed using the Agi4x44PreProcess Bioconductor package [13]. The original package was modified to apply LOESS normalization between arrays [14]. Subsequent data analysis was carried out using MATLAB (Mathwork, Inc., Massachusetts, USA). Probes that were identified as extreme outliers on the fold-change between replicate samples were removed from further analysis. The criterion for outlier detection was based on the inter-quantile range (IQR) method with α = 3 [15;16]. Finally, probes with low absolute expression levels were filtered out. The comparison of the remaining 23,457 probes for 8 cell samples is described further in the Results section.

## Results

### Enforced expression of E2A-PBX1 *in vivo* impairs differentiation in multiple hematopoietic lineages

In order to elucidate the impact of E2A-PBX1 on normal hematopoiesis, lethally-irradiated mice were transplanted with lin^-^ bone marrow cells that had been transduced with either control or E2A-PBX1-expressing retroviruses that co-express GFP. These animals were sacrificed three weeks after transplantation, before the emergence of any signs of illness, and cells from the bone marrow, spleen and thymus were isolated and analyzed.

**Fig 1:**
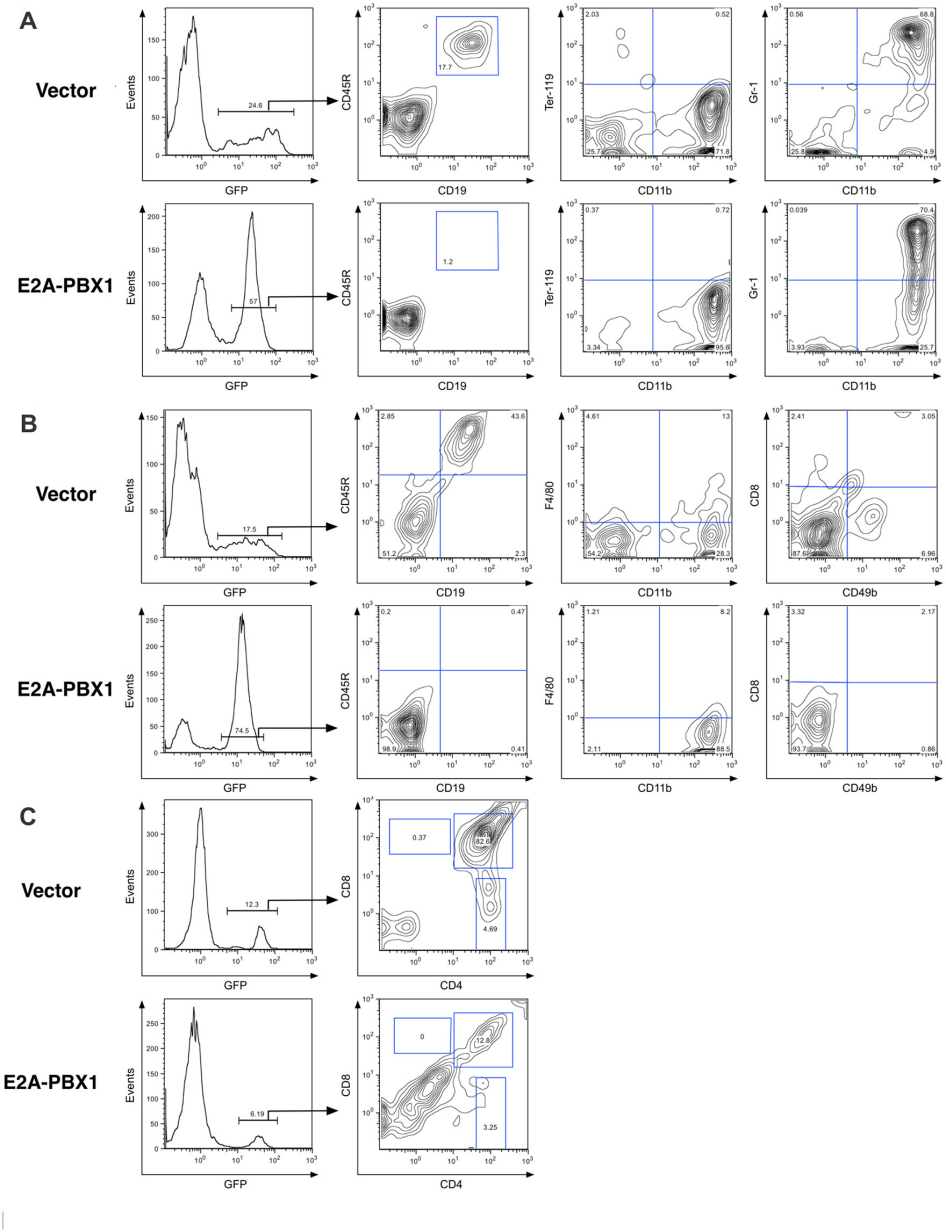
E2A-PBX1-expressing bone marrow cells reconstitute early myeloid but not B-lymphoid development in transplanted mice. Primary murine bone marrow cells were transduced with E2A-PBX1-expressing or control retroviral vectors and injected into the tail vein of lethally-irradiated, syngeneic recipient mice. Mice were sacrificed 3 weeks later. Immunophenotypic analysis of (A) bone marrow, (B) spleen, and (C) thymus are shown from representative animals. Events were gated so as to restrict all of these analyses to the GFP^+^ cell population.

E2A-PBX1-transduced cells failed to differentiate into committed CD45R^+^/CD19^+^ B-lymphoid progenitors; results from the bone marrow and spleen of a representative animal are shown in Fig 1A and 1B, respectively. No cells expressing surface IgM were observed among the GFP^+^ fraction in either tissue (data not shown). This was in contrast to control cells infected with the empty MIEV retroviral vector, in which CD45R^+^/CD19^+^ progenitors constituted 18% of cells in the bone marrow and fully 44% of cells in the spleen. A marked reduction in the number of CD49b^+^ cells in the spleen suggested impaired natural killer cell production. The thymus contained a reduced number of GFP^+^ cells expressing E2A-PBX1 relative to controls, consistent with impaired seeding of this organ due to impaired initiation of T-lymphopoiesis by E2A-PBX1-expressing progenitors (Fig 1C). Furthermore, co-staining for CD4 and CD8 showed a marked impairment in the ability of E2A-PBX1-expressing cells to mature beyond the double-negative stage. Equivalent results were observed in 4 animals transplanted with E2A-PBX1-transduced cells. Therefore, retrovirus-enforced expression of E2A-PBX1 in lin^-^ bone marrow-derived hematopoietic progenitors markedly impairs lymphoid development and, in particular, imposes an almost absolute block to B-lymphopoiesis.

Beyond the lymphoid compartment, there was impairment in maturation from CD11b^+^/Gr1^-^ to CD11b^+^/Gr1^+^ cells in the bone marrow and a reduction in F4/80^+^ macrophages in the spleen, suggesting a relative impairment of granulocytic/monocytic differentiation (Fig 1A and 1B). A modest reduction in Ter-119^+^ progenitors in the bone marrow suggested impaired erythroid differentiation (Fig 1A). Notwithstanding the evidence of impaired differentiation in multiple lineages, E2A-PBX1-expressing cells engrafted readily and accumulated in the bone marrow and spleen as an expanded population of CD11b^+^ cells. Therefore, enforced expression of E2A-PBX1 in this bone marrow transplantation model permits retention of granulocyte/monocyte lineage potential, albeit with a relative impairment of maturation.

### E2A-PBX1 antagonizes B-lymphoid differentiation *in vitro* through a mechanism that requires binding to both DNA and transcriptional co-activators

**Fig 2:**
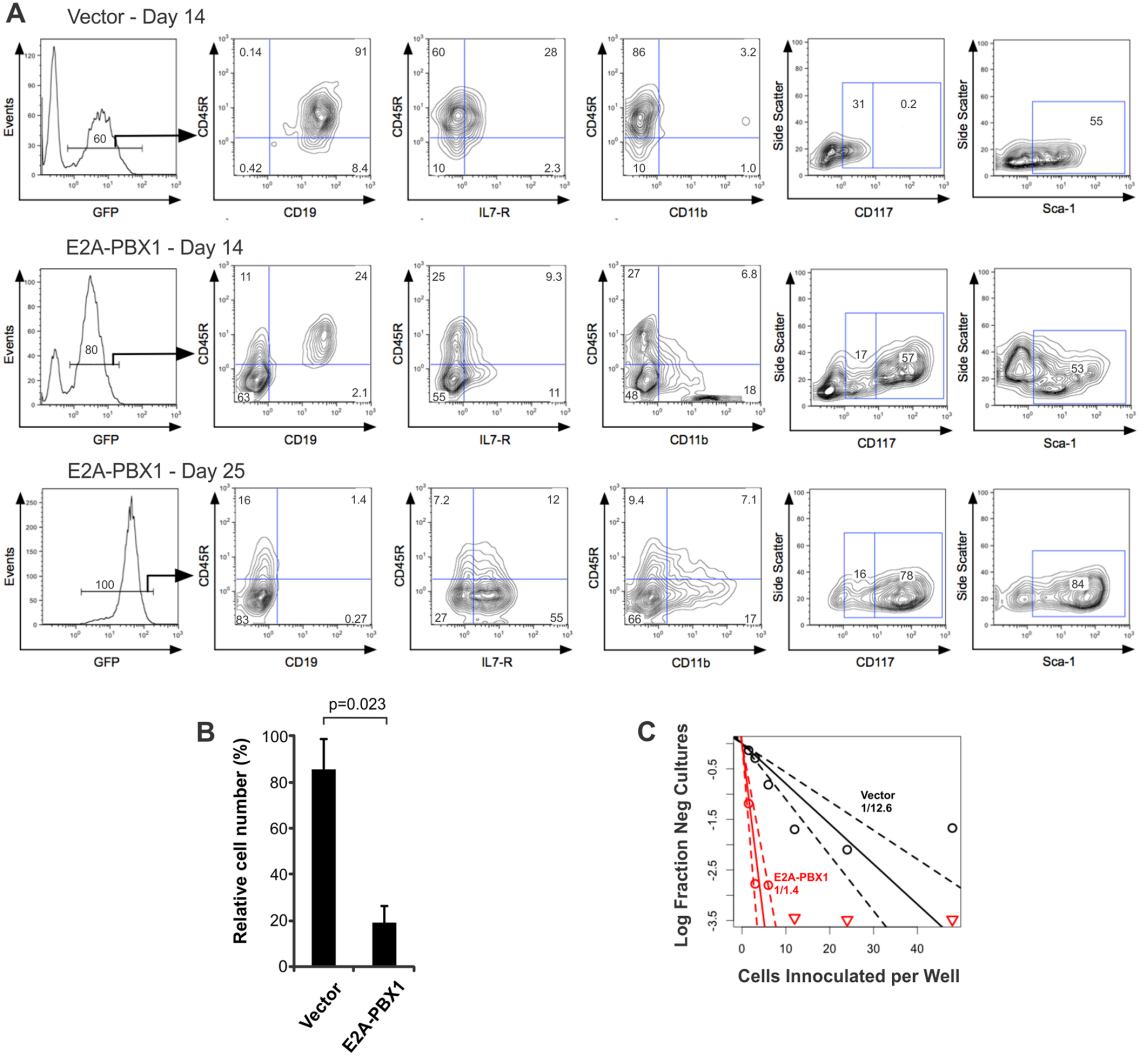
E2A-PBX1 blocks B-lymphoid differentiation *in vitro*. (A) Immunophenotype of E2A-PBX1-expressing FLPs after 14 days (top and middle rows) and 25 days (bottom row) of propagation in medium containing IL-7 and SCF. (B) Differential effects of SCF deprivation on E2A-PBX1-expressing FLPs. The bar graph shows the number of cells that were deprived of SCF expressed as a percentage of those that were maintained in SCF-containing medium. Data are from 2 independent experiments. Error bars represent one standard deviation. (C) Enumeration of culture-initiating cells by limiting dilution assays in 96-well plates. The slope of the solid line represents the log-active cell fraction and the dotted lines represent the 95% confidence intervals. Data points with no negative response are represented by down-pointing triangles. The calculated prevalence of culture-initiating cells is indicated on the plot.

In order to determine hematopoietic lineage fate, lineage-specific inductive signals must act upon multipotent progenitors that are competent to respond to them [17]. Therefore, the competency of E2A-PBX1-expressing, lineage-uncommitted progenitors to respond to environmental signals that normally induce B-lymphoid differentiation was investigated using an established, more experimentally tractable *in vitro* system. Lin^-^ hematopoietic progenitors harvested from day 12 to 14 fetal livers differentiate to committed pro-B-cells (also called “pre-B1 cells”) after 9 to 14 days of co-culture with OP9 stromal cells in the presence of IL-7 and SCF [18]. Whereas the immunophenotype of control cells confirmed their CD45R^+^/CD19^+^, B-lymphoid-committed, status (Fig 2A), most E2A-PBX1-transduced cells displayed a relatively immature, lineage-ambiguous phenotype characterized by the absence of CD45R or CD19, persistent expression of abundant CD117 and Sca-1, and variable expression of IL-7R and CD11b. Initially, minor populations of CD45R^+^/CD19^+^ (24%) and CD45R^+^/CD19^-^ (11%) cells were observed. While the CD45R^+^/CD19^+^ population was lost with continued culture (compare the CD45R/CD19 plots at 14 and 25 days), the CD45R^+^/CD19^-^ population was retained. The presence of these cells could reflect slight leakiness of the differentiation block or the presence at the time of retroviral transduction of a small number of lin^-^ progenitors with a bias towards the B-lymphoid fate. Equivalent results were obtained in two experiments. Despite its obvious effect on hematopoietic differentiation, expression of E2A-PBX1 did not affect growth kinetics during the first 14 days after retroviral transduction (S1 Fig). However, the ability of GFP^+^, E2A-PBX1-transduced cells to out-compete GFP^-^ cells by 25 days after transduction (Fig 2A) suggests the outgrowth of more proliferative subpopulations of cells many of which express E2A-PBX1 more abundantly. This is further supported by the right-ward shift in GFP signal intensity observed by day 25 for E2A-PBX1-transduced cells (Fig 2A). However, given the persistent expression of CD117 in E2A-PBX1-transduced FLPs, we considered whether the immature, E2A-PBX1-transduced cells were dependent on SCF, the ligand for CD117. Lin^-^ FLPs were deprived of SCF 5 days after transduction and the cumulative number of GFP^+^ cells was determined 9 days later. E2A-PBX1-transduced FLPs suffered a greater impairment of proliferation upon SCF deprivation than did control cells, indicating greater dependency on this cytokine (Fig 2B). Limiting dilution in 96-well plates and the web-based ELDA tool were used to determine the prevalence of culture-initiating cells. Whereas culture-initiating cells were present at a frequency of 1/12.6 cells among vector-infected FLPs, the frequency among E2A-PBX1-infected FLPs was nine-fold higher, at 1/1.4 (95% CI 1/17.5-1/9.06 versus 1/2.13-1/0.954, respectively, p < 0.001); Fig 2C) [12]. Equivalent results were obtained in three replicate experiments. The presence of features characteristic of early hematopoietic progenitors, including an immature immunophenotype, persistent differential responsiveness to SCF and a higher prevalence of culture-initiating cells in FLPs forced to express E2A-PBX1, supports the idea that E2A-PBX1 antagonizes aspects of hematopoietic maturation and enforces persistence of features characteristic of hematopoietic stem cells (HSCs) or early progenitors.

**Fig 3:**
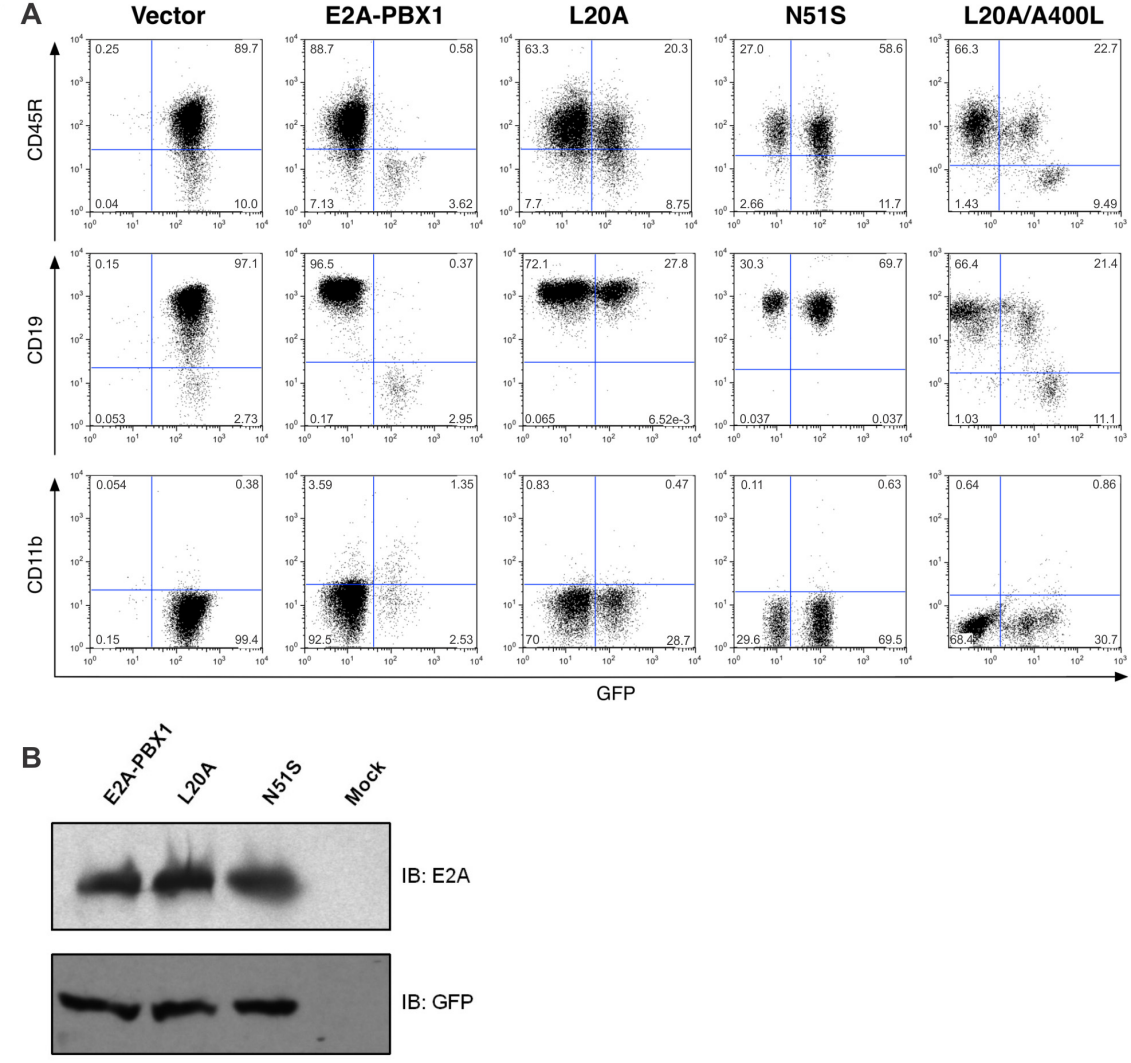
Interaction with both CBP/p300 and DNA is required in the E2A-PBX1-imposed differentiation blockade. (A) Immunophenotypic analysis of FLPs transduced with retroviruses conferring expression of E2A-PBX1 or the indicated amino acid-substituted mutants 14 days after transduction. Cells expressing unmodified E2A-PBX1, identified based upon GFP expression, fail to achieve B-lymphoid-committed, CD45R^+^/CD19^+^ status whereas this effect is lost consequent to the L20A or N51S amino acid substitutions that impair CBP/p300 recruitment or DNA binding, respectively. The ability of the L20A mutant protein to block B-lymphoid differentiation is partially restored by the A400L substitution, which increases the affinity of AD2 for CBP/p300. (B) Immunoblot of whole cell lysates from HEK293T cells transiently transfected with the indicated retroviral backbone plasmids. GFP serves as a control for transfection efficiency and gel loading. Expression of the L20A/A400L substituted E2A-PBX1 double-mutant at a level equivalent to that of un-modified E2A-PBX1 is documented elsewhere (22).

E2A-PBX1 induces proliferation in fibroblasts in culture or T-progenitor lymphoma in transgenic mice through a mechanism that does not require DNA binding mediated by the PBX1 homeodomain [3;19]. B-lymphoid commitment in the FLP model requires the *E2a* gene products [20]. These considerations raise the possibility that blocked B-lymphoid differentiation in our system could be attributable to dominant-negative effects on wild-type *E2a* gene products, perhaps involving squelching through sequestration of transcriptional co-activators by E2A-PBX1. The L20A amino acid substitution within activation domain 1 (AD1) of E2A-PBX1 markedly impairs binding to the transcriptional co-activator and histone acetyltransferase CBP/p300 whereas the N51S substitution in the PBX1 homeodomain disrupts DNA binding [5;21]. Either of these substitutions completely abrogated the differentiation blockade imposed by E2A-PBX1 in the FLP-based B-lymphopoiesis model (Fig 3A). The loss of the differentiation block was not due to sub-threshold transgene expression as both the L20A and N51S mutants are expressed at levels comparable to un-modified E2A-PBX1 (Fig 3B). These results, which were obtained in at least two independent experiments, support a model in which E2A-PBX1 blocks B-lymphoid differentiation through a mechanism that requires the recruitment of CBP/p300 to genomic loci bound by the PBX1 homeodomain.

We recently used NMR spectroscopy to demonstrate binding of activation domain 2 (AD2) of E2A to the KIX domain of CBP and determined that an A400L substitution within AD2 increases the affinity of this interaction by more than two-fold (dissociation constant (*K*_d_) changes from 17 to 7.2 μM) [22]. Addition of the A400L substitution to full-length, L20A-substituted E2A-PBX1 so as to create an L20A/A400L double-mutant partially reconstituted the differentiation block that was lost as a result of the L20A substitution alone (Fig 3A) such that cells emitting brighter GFP fluorescence, and therefore presumably expressing more E2A-PBX1 L20A/A400L, failed to penetrate into the B-lymphoid compartment while cells with dimmer GFP fluorescence succeeded in achieving B-lymphoid commitment. This effect is attributable to the A400L substitution as the L20A/A400L double-mutant is expressed at levels equivalent to wild type E2A-PBX1 and the L20A single mutant [22]. These results suggest that AD2 can supplant defective AD1 in recruiting CBP/p300 to DNA-bound E2A-PBX1 and that this is marginally sufficient for blocking differentiation. More generally, these results provide additional support for the importance of CBP/p300 recruitment in mediating the differentiation blockade imposed by E2A-PBX1.

### E2A-PBX1-transduced FLPs can undergo myeloid differentiation in response to GM-CSF

**Fig 4:**
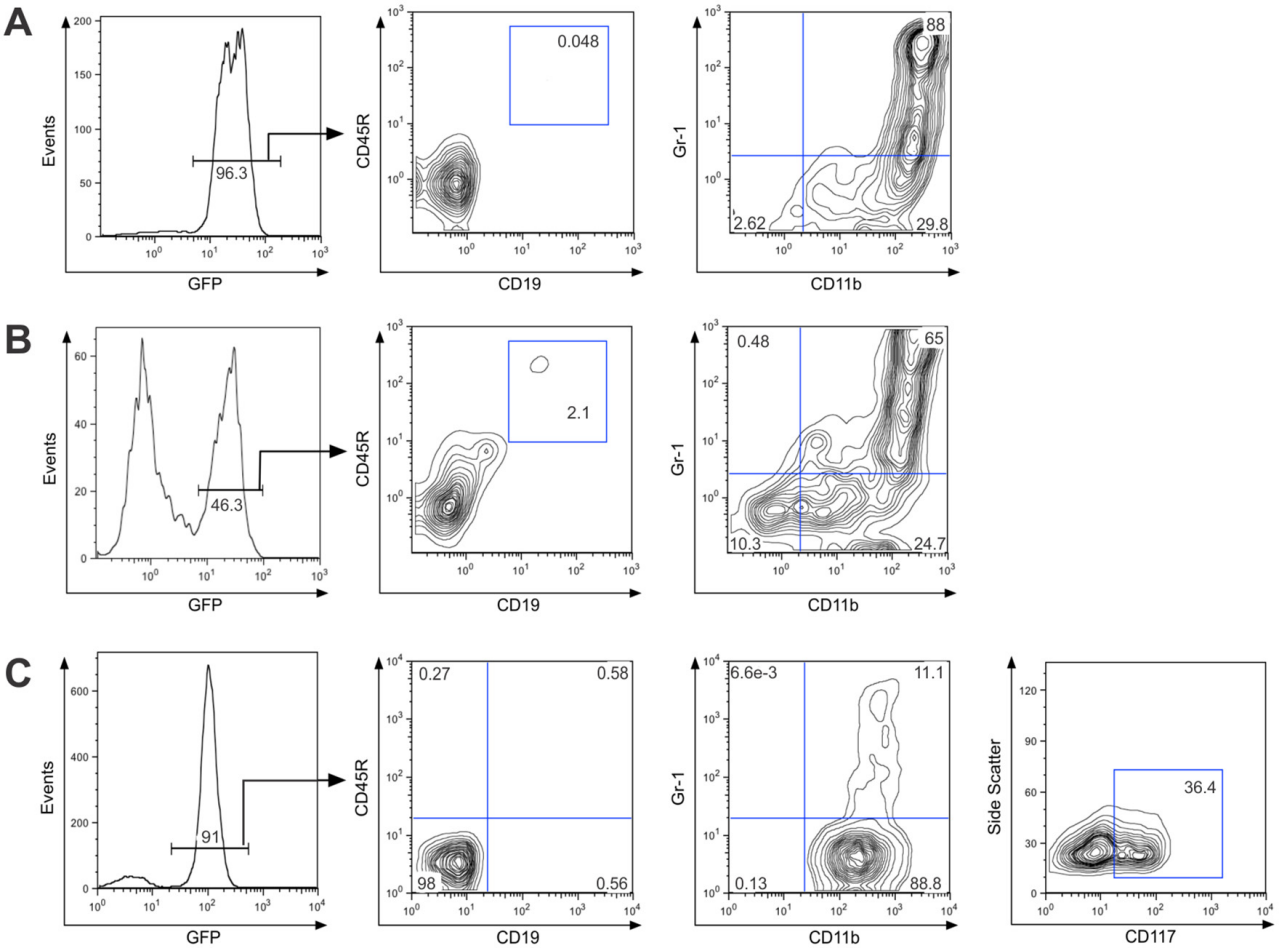
FLPs expressing E2A-PBX1 remain amenable to induction of myeloid differentiation *in vivo* or *in vitro*. E2A-PBX1-transduced FLPs were injected into the tail veins of lethally-irradiated mice. Immunophenotypic analysis was performed on cells obtained from the bone marrow (A) or spleen (B) 3 weeks later. (C) E2A-PBX1 expressing FLPs were transferred from medium containing IL-7 and SCF to medium containing GM-CSF instead and analyzed by flow cytometry 7 days later.

Although the vast majority of E2A-PBX1-expressing FLPs cultured with IL-7 and SCF remain relatively undifferentiated (Fig 2A), bone marrow progenitors overwhelmingly adopt a CD11b^+^ myeloid immunophenotype after transplantation (Fig 1A and 1B), suggesting that E2A-PBX1-expressing progenitors remain responsive to pro-myeloid stimuli provided in the bone marrow or splenic microenvironments. This possibility was investigated using the FLP model by transplanting cultured E2A-PBX1-transduced FLPs into lethally-irradiated recipient mice. These rapidly developed leukemia; all three animals succumbed to the disease by 38 days post-transplantation. One mouse was euthanized at 21 days post-transplantation, prior to manifesting signs of illness. Immunophenotypic analysis of bone marrow (Fig 4A) and spleen (Fig 4B) from this animal revealed that the vast majority of GFP^+^ cells were CD11b^+^, indicating myeloid differentiation. This observation was replicated *in vitro*; 14 days after transduction, E2A-PBX1-expressing FLPs were transferred from media containing IL-7 and SCF to media in which the only exogenously added recombinant cytokine was murine granulocyte/macrophage colony stimulating factor (GM-CSF). Compared to E2A-PBX1-expressing FLPs cultured in the presence of IL-7 and SCF, most of which were CD11b^-^, CD117^+^ (Fig 2A), the vast majority of cells cultured in GM-CSF acquired a myeloid immunophenotype reminiscent of that of the GFP^+^ cells that accumulated in the bone marrow of transplanted mice: CD11b^+^, Gr-1^+^, CD117^+/-^, CD45R^-^, CD19^-^ and IL-7R^-^ (Fig 4C and data not shown). Equivalent results were obtained in two independent experiments. Therefore, the results obtained using our FLP-based model agreed with those based on transplantation of bone marrow progenitors in showing that uncommitted progenitors that express E2A-PBX1 become leukemogenic and manifest a dense block to B-lymphoid commitment while remaining amenable to the induction of granulocytic/monocytic differentiation.

### E2A-PBX1 blocks B-lymphopoiesis prior to the common lymphoid progenitor stage

**Fig 5:**
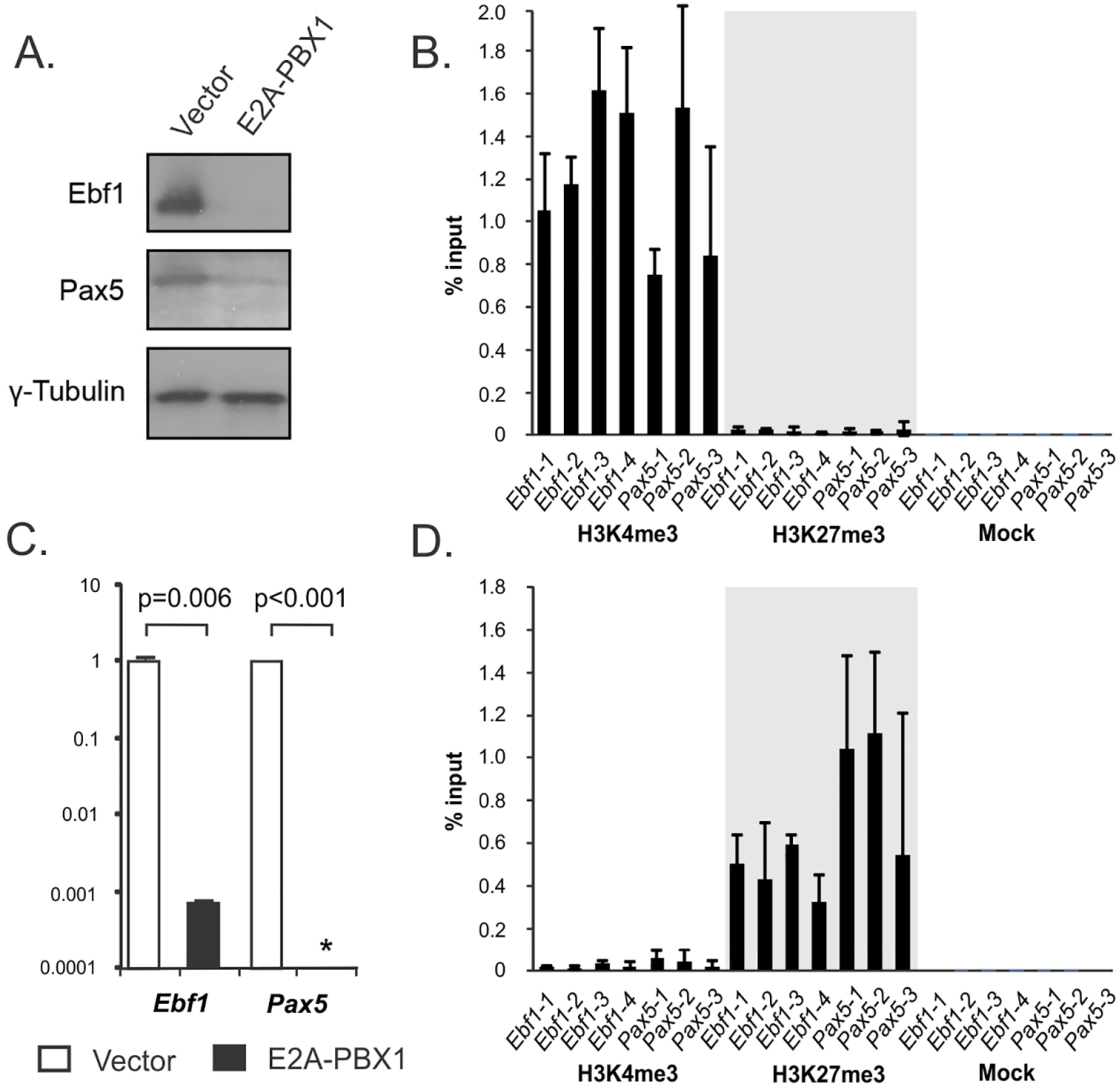
B-lymphopoiesis is blocked at a point prior to the CLP stage in FLPs expressing E2A-PBX1. (A) Immunoblotting indicates markedly reduced or absent expression of the B-lymphopoietic transcription factors Ebf1 and Pax5 in FLPs expressing E2A-PBX1 relative to those infected with the empty retroviral vector. The apparent weak signal for Pax5 in the E2A-PBX1 lane may represent spillover between lanes. Anti-γ-tubulin serves as a loading control. (B) ChIP-qPCR results obtained from chromatin prepared from control, vector-infected FLPs indicate prevalence of the activating H3K4me3 mark relative to the silencing H3K27me3 mark at the *Ebf1* and *Pax5* promoters. Error bars represent 95% confidence limits. (C) Results from qRT-PCR indicating marked reduction or absence of the Ebf1 and Pax5 transcripts in FLPs expressing E2A-PBX1 relative to control cells. The asterisk indicates failure to observe a PCR product in either of the 2 independent samples. Error bars indicate one standard deviation. (D) ChIP-qPCR results obtained from chromatin prepared from FLPs expressing E2A-PBX1 indicate prevalence of the silencing H3K27me3 mark relative to the activating H3K4me3 mark at the *Ebf1* and *Pax5* promoters.

During B-lymphopoiesis, the progeny of HSCs undergo progressive restriction of their lineage potential through the multipotent progenitor (MPP), lymphoid-primed MPP and common lymphoid progenitor (CLP) stages [23]. Commitment to the B-lineage occurs subsequently at the pro-B-cells stage, which coincides with surface expression of CD19. The B-lymphopoietic transcription factor genes *Ebf1* and *Pax5* are subjected to Polycomb group- (PcG) mediated silencing in HSCs and MPPs but are transcriptionally induced when cells reach the CLP stage [11]. Accordingly, the *Ebf1* and *Pax5* promoters are associated with the PcG-mediated repressive mark H3K27me3 in early hematopoietic and non-lymphoid progenitors, whereas they acquire the activating H3K4me3 mark in CLPs. Evaluating the E2A-PBX1-expressing FLPs indicated low to absent expression of *Ebf1* or *Pax5* at either the protein or transcript levels (Fig 5A and 5C). Examining the chromatin status at the *Ebf1* and *Pax5* promoters by ChIP-qPCR demonstrated failure of either promoter to switch from the silenced H3K27me3- to the active H3K4me3-marked state in E2A-PBX1-expressing FLPs (Fig 5B and 5D). Therefore, B-lymphopoiesis is blocked prior to the CLP stage as a consequence of E2A-PBX1 expression.

### Forced expression of E2A-PBX1 deregulates hematopoietic genes

**Fig 6:**
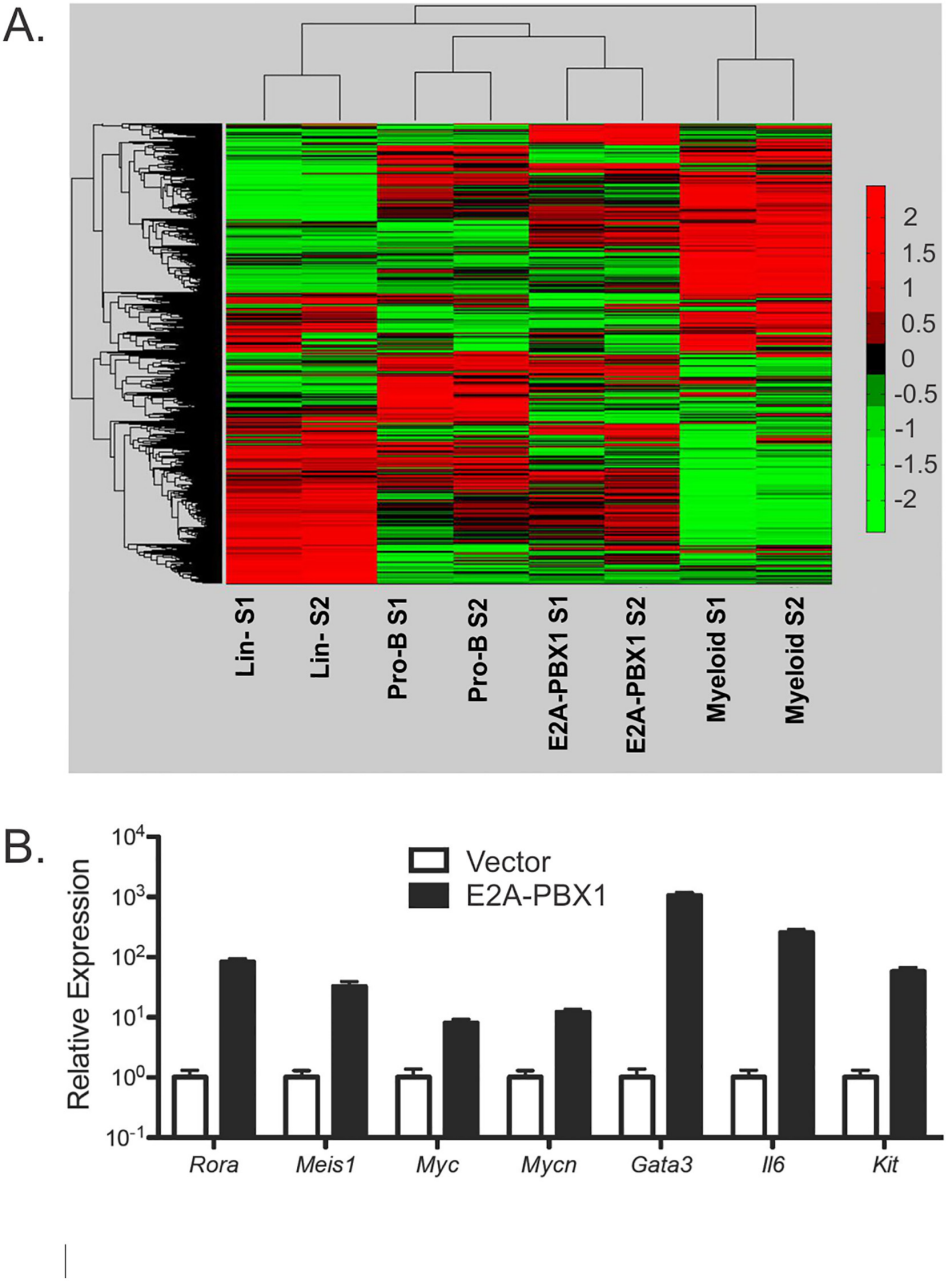
Forced expression of E2A-PBX in FLPs deregulates hematopoietic genes. (A) Hierarchical cluster analysis performed on expression data from gene expression microarrays indicates that the closest relationship to E2A-PBX1-expressing FLPs is with pro-B-cells that were differentiated from FLPs *in vitro*. (B) Confirmation by qRT-PCR of differential expression of genes identified initially using expression microarrays in E2A-PBX-expressing versus pro-B-cells derived from vector-infected FLPs. Data are from 2 independent samples. Error bars indicate one standard deviation; p-values are less than 0.005 for each comparison.

Gene expression microarray analysis was used to further elucidate the effects of E2A-PBX1 on hematopoiesis. RNA from biological replicates of E2A-PBX1-transduced FLPs and vector-transduced, FLP-derived, CD19^+^ pro-B-cells was evaluated for differential gene expression. The immunophenotype of the E2A-PBX1-tranduced cells used for gene expression analysis was equivalent to that shown in Fig 2A for day 25. RNA prepared from primary, uncultured lin^-^ FLPs and primary CD11b^+^ myeloid bone marrow cells was evaluated for comparison with the cultured cells. Hierarchical cluster analysis based on gene expression profiles of all of the probes that remained after initial data filtering (n = 23,457) showed that the FLP-derived pro-B-cells and the FLP-derived E2A-PBX1-expressing cells clustered together (Fig 6A). This is consistent with the common origin of these cell types from lin^-^ FLPs and with their history of having been maintained in tissue culture for several passages. Further analysis of the data identified 3,203 probes representing 2,235 genes that showed at least a four-fold difference in expression between E2A-PBX1-expressing cells and FLP-derived pro-B-cells; these are listed in S2 Table. In S3 Table, these genes are organized according to their expression in lin^-^ or myeloid cells. For example, abundant expression of *Mycn*, *Il6*, *Bcl2*, *Flt3* and *Hoxa9* was exclusive to E2A-PBX1-expressing FLPs, whereas abundant *Rora*, *Meis1*, *Gata 2* and *Gata3* was observed in both E2A-PBX1-expressing and lin^-^ FLPs. Differential up-regulation of *Rora*, *Meis1*, *Myc*, *Mycn*, *Gata3*, *Il6* and *Kit* in E2A-PBX1-expressing relative to FLP-derived pro-B-cells was confirmed by qRT-PCR (Fig 6B). Enrichment analysis for Kyoto Encyclopedia of Genes and Genomes (KEGG) pathways was performed using the Database for Annotation, Visualization and Integrated Discovery (DAVID) [24]. The top 20 KEGG pathways over-represented by genes up- or down-regulated downstream of E2A-PBX1 are listed in Tables 1 and 2, respectively; the full lists of pathways and the probe ID numbers of the genes associated with each pathway are provided in S4 and S5 Tables. Genes up-regulated in E2A-PBX1-expressing cells and over-represented from the "Cytokine-cytokine receptor interaction" pathway included many genes encoding cytokines or cytokine receptors, such as *Csf1r*, *Flt1*, *Flt3*, *Met*, *Hgf*, *Pdgfc*, *Vegfc*, while genes over-represented from the "Hematopoietic cell lineage" pathway included genes characteristically expressed in myeloid cells and progenitors, such as *Cd14*, *Cd33*, *Cd36*, *Gp1ba* and *Gp1bb*. Genes down-regulated in E2A-PBX1-expressing cells and over-represented from the "Pathways in cancer" pathway included known tumor suppressor genes, such as *Cdkn1b*, *Pten* and *Foxo1*; genes over-represented from the "B cell receptor signaling pathway" included those encoding signaling molecules and transcription factors such as *Cd19*, *Cd79a*, *Cd79b*, *Blnk*, *Nfkb1a* and *Tcf3*. These patterns of gene expression are consistent with failure of E2A-PBX1-expressing FLPs to commit to the B-lymphoid lineage, their retention of myeloid potential and their proliferative and leukemogenic properties.

**Table 1:** KEGG pathways overrepresented by genes up-regulated downstream of E2A-PBX1.

**KEGG** p**athway** t**erm**	Number of genes	*P* value
Cytokine-cytokine receptor interaction	76	4.34E-13
Hematopoietic cell lineage	33	9.53E-09
Cell adhesion molecules	44	1.05E-06
Lysosome	34	2.22E-05
Intestinal immune network for IgA production	20	3.75E-05
Graft-versus-host disease	20	1.14E-04
Dilated cardiomyopathy	27	1.16E-04
Glycolysis / Gluconeogenesis	22	1.32E-04
ECM-receptor interaction	24	3.88E-04
Hypertrophic cardiomyopathy	24	4.69E-04
Leukocyte transendothelial migration	30	7.67E-04
Chemokine signaling pathway	41	8.24E-04
Jak-STAT signaling pathway	35	0.0014
Arrhythmogenic right ventricular cardiomyopathy	21	0.0015
Glutathione metabolism	16	0.0025
Asthma	12	0.0026
Type I diabetes mellitus	18	0.0029
Allograft rejection	17	0.0030
Pentose phosphate pathway	10	0.0048
Natural killer cell mediated cytotoxicity	28	0.0050

**Table 2:** KEGG pathways overrepresented by genes down-regulated downstream of E2A-PBX1.

**KEGG** p**athway** t**erm**	Number of genes	*P* value
Focal adhesion	47	1.23E-08
Axon guidance	31	6.24E-06
Pathways in cancer	56	2.23E-05
B cell receptor signaling pathway	21	5.98E-05
ECM-receptor interaction	21	1.04E-04
Small cell lung cancer	21	1.49E-04
Colorectal cancer	21	1.76E-04
Hematopoietic cell lineage	20	3.72E-04
Base excision repair	12	0.0011
Primary immunodeficiency	11	0.0018
Wnt signaling pathway	27	0.0024
Vascular smooth muscle contraction	23	0.0027
MAPK signaling pathway	41	0.0033
Mismatch repair	8	0.0038
Drug metabolism	16	0.0053
Adherens junction	16	0.0061
Histidine metabolism	8	0.0083
Glutathione metabolism	12	0.0105
Glycerolipid metabolism	11	0.0138
VEGF signaling pathway	15	0.0144

### Ectopic expression of *Hoxa9*, but not *Bmi1*, antagonizes B-lymphoid differentiation *in vitro*

**Fig 7:**
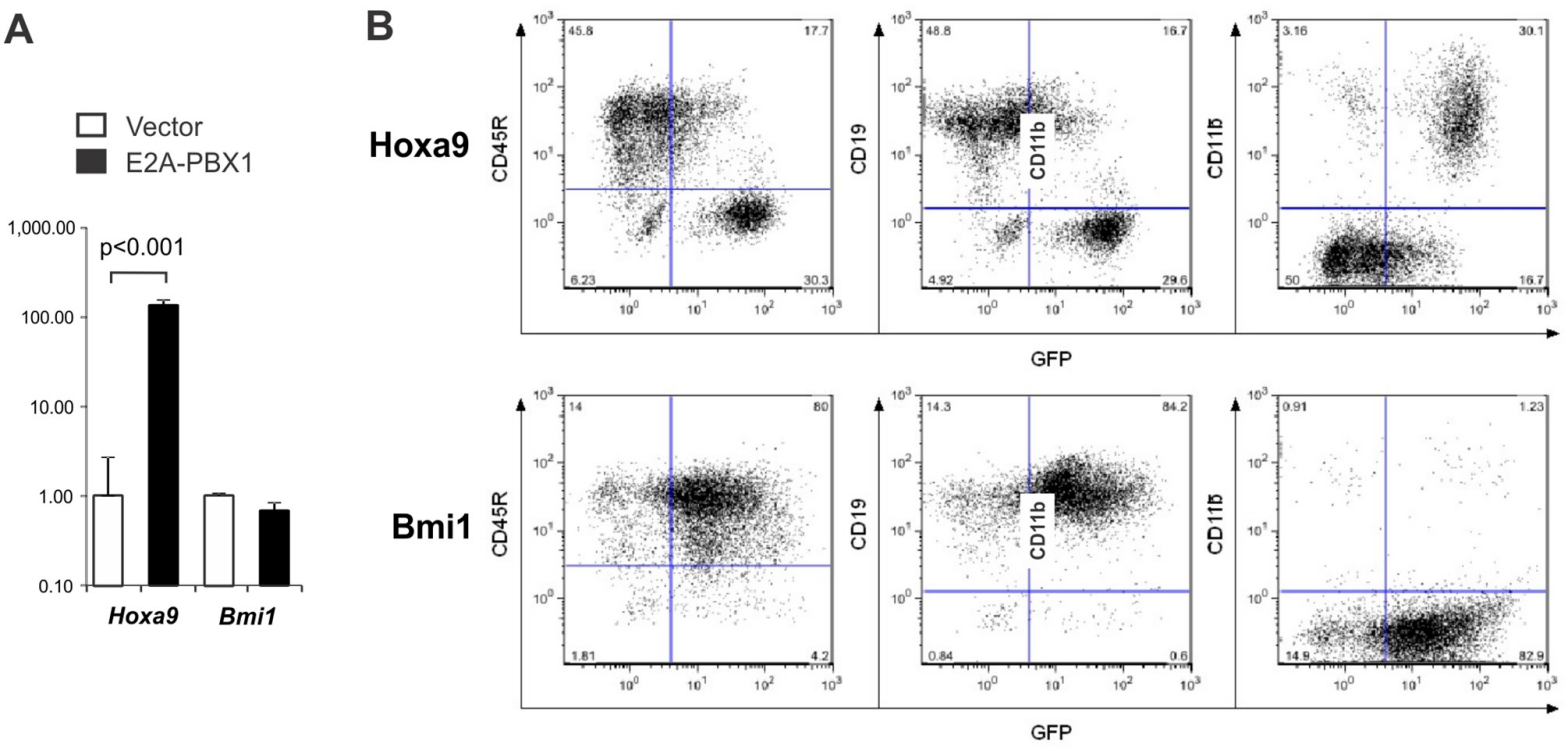
Ectopic expression of *Hoxa9*, but not *Bmi1*, in fetal liver progenitors antagonizes B-lymphoid differentiation. (A) Analysis by qRT-PCR of *Hoxa9* and *Bmi1* gene expression in E2A-PBX1- versus vector-infected FLPs. Values for each gene are expressed relative to expression in vector-infected cells. (B) Immunophenotype of FLPs transduced with *Hoxa9*- versus *Bmi1*-expressing retroviruses. Lin^-^ FLPs were transduced with retroviruses expressing *Bmi1* or *Hoxa9* and then cultured under B-lymphoid conditions. Cells were analyzed by flow cytometry on day 14 post-infection.

Of the differentially regulated genes uncovered by expression analysis, *Hoxa9* is an especially attractive candidate as a mediator of E2A-PBX1-induced hematopoietic effects in our experiments. *Hoxa9* is expressed abundantly in HSCs and early progenitors and subsequently down-regulated in more mature progenitors [25]. Abundant expression of *HOXA9* in clinical samples of acute myelogenous leukemia is associated with adverse clinical outcomes [26]. Enforced expression of *Hoxa9* in murine hematopoietic progenitors antagonizes lymphopoiesis and, in collaboration with *Meis1*, induces myeloproliferative neoplasia [27–29]. Our microarray analysis indicated 40-fold higher expression of *Hoxa9* in E2A-PBX1-expressing FLPs relative to vector-infected pro-B-cells (S2 Table) whereas qRT-PCR applied to independent cell preparations indicated 134-fold higher expression (Fig 7A). Accordingly, FLPs were transduced with a *Hoxa9* retrovirus and grown under B-lymphopoietic conditions in order to investigate whether enforced expression of *Hoxa9* could replicate the phenotype produced by E2A-PBX1. Flow cytometry carried out 14 days after transduction demonstrated that Hoxa9 blocked B-lymphoid commitment in what appeared to be a dose-dependent manner; whereas GFP-dim cells that presumably produced Hoxa9 less abundantly succeeded in achieving CD19^+^, CD11b^-^ pro-B-cell status, GFP-bright cells expressing more abundant Hoxa9 instead manifested a CD19^-^, CD11b^+^ phenotype suggestive of myeloid differentiation (Fig 7B). The PcG protein Bmi1 normally silences the B-lymphopoietic transcription factor genes *Ebf1* and *Pax5* in early, lineage-uncommitted progenitors and has been implicated as a mediator of E2A-PBX1-driven oncogenesis [11;30]. However, the Bmi1 transcript was not over-expressed in E2A-PBX1-expressing FLPs and retrovirus-enforced expression of Bmi1 failed to block B-lymphoid commitment (Fig 7A and 7B). These results support the notion that induction of *Hoxa9*, but not *Bmi1*, is involved in mediating E2A-PBX1-dependent antagonism of B-lymphopoiesis in our experimental model.

### E2A-PBX1 induces cell cycle arrest and apoptosis in committed B-lymphoid progenitors

**Fig 8:**
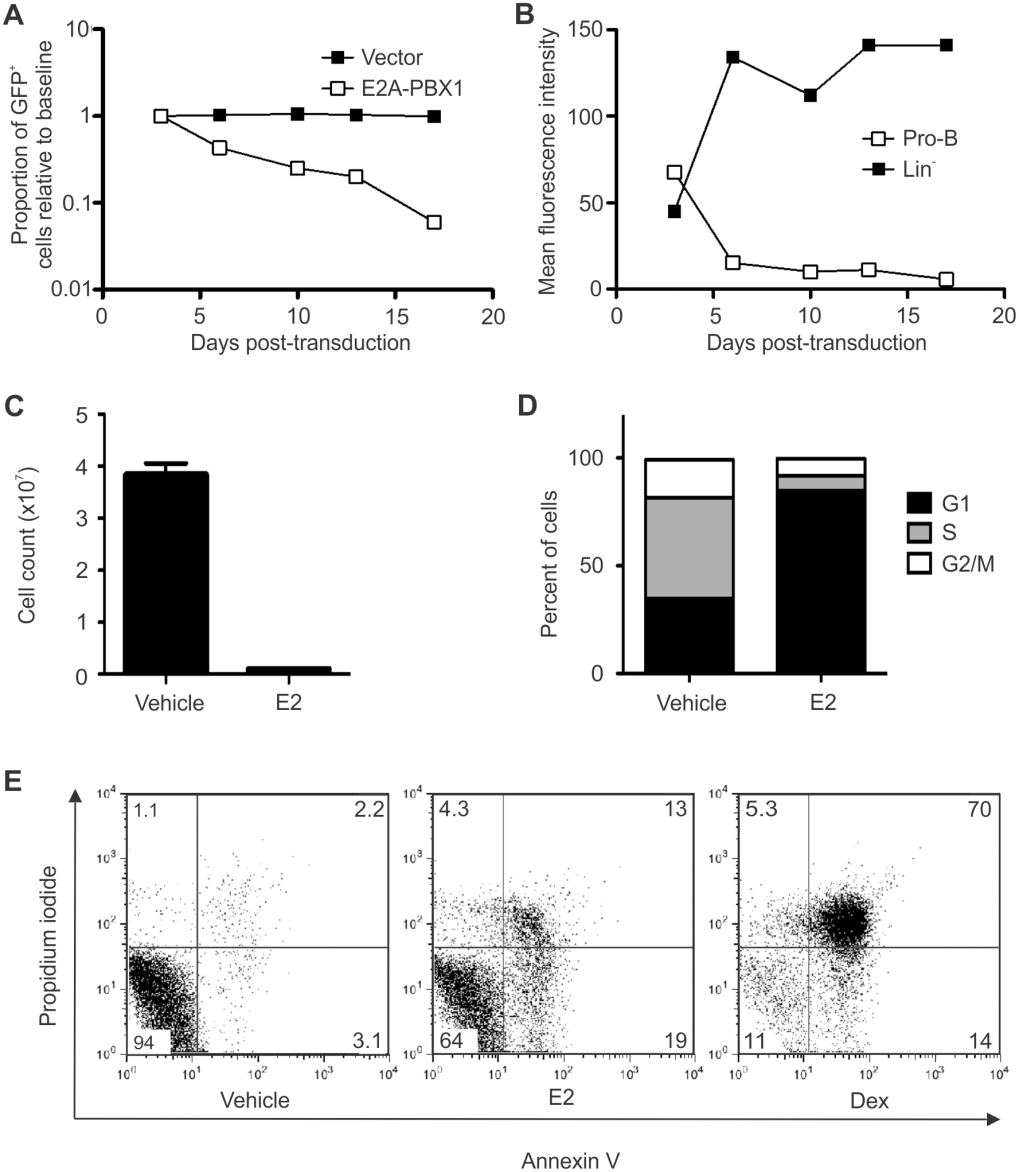
E2A-PBX1 induces cell cycle arrest and apoptosis in committed B-lymphoid progenitors. (A) FLP-derived pro-B-cells were infected with GFP-expressing retroviral vectors and the proportion of GFP^+^ cells was followed over time by flow cytometry. The graph shows the proportion of GFP^+^ cells relative to the value determined on day 3 after each transduction. (B) FLP-derived pro-B-cells versus lin^-^ FLPs were infected with a retroviral vector that co-expresses GFP and E2A-PBX1 and then the mean fluorescence intensity (MFI) amongst GFP^+^ cells was followed over time by flow cytometry. (C) 3PER12 cells, pre-B1-derived cells that stably express EPΔ623ER, an engineered, estrogen-regulatable variant of E2A-PBX1, were induced with 10 μM estradiol (E2) versus ethanol alone (vehicle) and the accumulation of viable, Trypan blue-excluding cells over the next 72 hours was determined. (D) Flow cytometry-based cell cycle analysis of 3PER12 cells after 72 hours of induction with E2 versus vehicle. (E) Flow cytometry-based detection of apoptosis in 3PER12 cells. Cells in early apoptosis (annexin V^+^, propidium iodide^-^) or late apoptosis or death (annexin V^+^, propidium iodide^+^) are more numerous after E2 induction. Cells treated with dexamethasone (Dex) serve as a positive control.

In clinical samples of t(1;19)-positive ALL, the presence of random nucleotides at the fusion point between *E2A*- and *PBX1*-derived exons and the tight clustering of breakpoints in the *E2A* locus suggest that the translocation may be mediated by the recombinase machinery that normally mediates immunoglobulin gene rearrangements [31]. Thus, the pre-B-cell phenotype typically observed in cases of t(1;19)-associated ALL could reflect occurrence of the leukemia-initiating translocation event in a committed B-lymphoid progenitor. In an attempt to model this leukemogenic event, FLP-derived pro-B-cells were transduced with an E2A-PBX1-expressing retroviral vector and then monitored for GFP prevalence by flow cytometry (Fig 8A). The prevalence of E2A-PBX1/GFP-expressing cells declined over time in culture, indicating that E2A-PBX1 exerts an anti-proliferative effect; this experiment was carried out twice with equivalent results (data not shown). Next, mean fluorescence intensity (MFI) for GFP was followed over time in FLP-derived pro-B-cells versus lin^-^ FLPs after transduction with a E2A-PBX1/GFP-expressing retrovirus (Fig 8B). In lin^-^ FLPs the MFI increased over time, consistent with the results shown in Fig 2A and suggesting that clones expressing abundant E2A-PBX1 derive a proliferative advantage. In contrast, the MFI amongst pro-B-cells declined over time, implying selection against clones expressing higher levels of E2A-PBX1. Equivalent results were obtained in multiple experiments. In order to investigate the mechanism by which E2A-PBX1 exerts its anti-proliferative effects we developed a system in which the function of E2A-PBX1 could be regulated in committed murine B-lymphoid progenitors. "Pre-B-1" cells from the bone marrow of adult mice were isolated, expanded *ex vivo* according to an established protocol [8], and rendered stromal cell- and IL-7-independent by stable, retrovirus-mediated expression of p210^BCR-ABL^. These cells were then transduced with a retrovirus conferring expression of EPΔ623ER, an estrogen-dependent derivative of E2A-PBX1 [32]; the resulting CD19^+^ cell line was denoted 3PER12. 3PER12 cells cultured for 72 hours in the presence of estradiol (E2) appeared small and refractile, suggesting the occurrence of widespread apoptosis, whereas control cells treated with vehicle alone, or the parent cell line treated with E2, appeared unaffected (S2 Fig and data not shown). Counts of viable cells demonstrated markedly reduced proliferation over 72 hours (Fig 8C) and cell cycle analysis determined that the majority of cells treated with E2 were arrested in G1 (Fig 8D). In comparison with vehicle-treated controls, 3PER12 cells treated with E2 displayed a marked increase in apoptosis (Fig 8E). These results indicate that, whereas E2A-PBX1 is demonstrably oncogenic when introduced into uncommitted murine hematopoietic cells, when expressed in committed B-lymphoid progenitors it induces cell cycle arrest and apoptosis.

## Discussion

The availability of an experimentally tractable murine model in which enforced expression of E2A-PBX1 gives rise to B-lymphoid neoplasia would greatly advance efforts to elucidate the molecular mechanisms involved in E2A-PBX1-driven ALL. Accordingly, much of the impetus for the current study arose from a desire to understand the observation that mice transplanted with E2A-PBX1-tranduced bone marrow succumb mostly to myeloid rather than B-lymphoid neoplasms [5;6;33]. We also considered that elucidating differential effects of E2A-PBX1 expression on hematopoietic progenitors of diverse lineages would provide insights into E2A-PBX1 function that would inform future, more directly mechanistic studies. Our findings indicate that, when expressed in uncommitted progenitors, E2A-PBX1 antagonizes aspects of both myeloid and lymphoid maturation and enforces the persistence of phenotypic features characteristic of HSCs or early progenitors. Our results also demonstrate differential E2A-PBX1-mediated effects on lymphoid relative to myeloid differentiation: whereas impairment of myeloid differentiation is merely relative such that it permits the expansion of leukemogenic myeloid progenitors in a myeloid-inductive local environment, the block to B-lymphopoiesis is near-absolute. Furthermore, in contrast to the selective advantage it confers when introduced into uncommitted progenitors, E2A-PBX1 causes cell cycle arrest and apoptosis in committed B-lymphoid progenitors. Therefore, our results demonstrate that the phenotype conferred by E2A-PBX1 is critically dependent on the maturity and lineage of the hematopoietic cells into which it is introduced.

Several recent findings allow us to speculate as to how E2A-PBX1 might perturb hematopoiesis when it is introduced into lin^-^ progenitors. Lineage specification is governed to an important degree by the lineage-specific activity of enhancers [34]. Wild-type PBX1 has been shown to bind DNA in a manner influenced by the presence of specific chromatin marks, and the leukemogenic transcription factors PML-RARα and AML1-ETO were shown recently to bind preferentially to enhancers that are associated with accessible chromatin and local binding by p300 [35;36]. Our results suggesting that E2A-PBX1 must bind to DNA by means of the PBX1-derived homeodomain in order to abrogate the lymphoid lineage potential of uncommitted progenitors (Fig 3) are consistent with previous results indicating that the PBX1-derived homeodomain is required for impeding myeloid differentiation by E2A-PBX1 [19;37]. Therefore, it is reasonable to suggest that, upon its introduction into uncommitted progenitors, E2A-PBX1 binds regulatory elements through a mechanism that involves the PBX1 homeodomain and in a genomic distribution that is determined in part by the status of local chromatin. It may then associate with transcriptional co-regulators including CBP/p300 and perturb enhancer function, thereby impairing normal maturation and promoting the expansion of progenitors that remain responsive to myeloid-inductive environmental stimuli but blocked in a proliferative stage of development. Hematopoietic lineage specification is associated with major alterations in the accessibility of the chromatin at, in particular, lineage-specific enhancers and a progressive, overall decrease in chromatin accessibility [38]. Thus, the striking effect of the stage of hematopoietic cell differentiation that we have demonstrated on the phenotypic consequences of E2A-PBX1 expression could reflect the influence of different chromatin states in determining the genomic location and downstream transcriptional consequences of E2A-PBX1 binding.

In contrast to its proliferative and oncogenic effects in lin^-^ FLPs (Figs 1 and 2), we demonstrate that forced expression of E2A-PBX1 in FLP-derived pro-B-cells induced cell cycle arrest and apoptosis (Fig 8). The ability of E2A-PBX1 to induce apoptosis in B-lymphoid progenitors has been reported previously, albeit not in FLP-derived cells [39;40]. The distinctive response of committed B-lymphoid cells to E2A-PBX1 could reflect a cellular response to oncogene activation. For example, the ability of p185^BCR-ABL^ to induce murine B-lineage ALL, but not myeloid neoplasia, is enhanced dramatically by inactivation of the *Arf* tumor suppressor gene [41]. *Arf* lies within the *Cdkn2a* locus and its product, p19^Arf^, can induce p53-mediated cellular senescence or apoptosis in response to oncogene activation [42]. The differential susceptibility of FLP-derived pro-B-cells to E2A-PBX1-induced apoptosis and cell cycle arrest could reflect the distinctive status of *Cdkn2a* in these cells; whereas this locus is epigenetically silenced in HSCs, it is poised for induction in committed B-progenitors [41]. In support of the existence of an antagonistic relationship between E2A-PBX1 and *CDKN2A* in the clinical context, deletions affecting *CDKN2A* at chromosome band 9p21 are exceptionally prevalent amongst human ALL samples associated with t(1;19) [43]. More generally, our results suggest that additional oncogenic alterations, such as inactivation of tumor suppressors or activation of pro-survival oncogenes, may be required for the oncogenic effects of E2A-PBX1 to be manifested in in B-lymphoid cells. Other possible explanations for the failure of E2A-PBX1 to impart a selective growth advantage in our FLP-derived progenitors include possible deficiencies in the specific culture conditions that we provided relative to the requirements of E2A-PBX1-expressing B-lymphoid cells, failure to target E2A-PBX1 expression to a specific, more permissive stage of B-lymphoid ontogeny, and inherent differences between human and murine progenitors.

Sykes and Kamps used an approach similar to our own to investigate how the hematopoietic fate of bone marrow cells is affected by retrovirus-driven expression of E2A-PBX1 [44]. These authors determined that unfractionated mononuclear bone marrow cells infected with an E2A-PBX1 retrovirus and then cultivated in the presence of SCF and IL-7 developed into early T-lymphoid progenitors, whereas control cells developed into CD19-expressing pro-B-cells. Although methodological differences, including the use of bone marrow- versus FLP-derived progenitors for *in vitro* experiments, impede direct comparison of this study with our own, it is noteworthy that, whereas the evidence supporting T-lymphoid lineage was derived exclusively from *in vitro* data, our current study is the first to include transplantation experiments in which the lineage potential of E2A-PBX1-expressing lin^-^ hematopoietic cells was determined directly in reconstituted mice. Instead of an expansion of T-lymphoid progenitors, we demonstrate profound defects in thymic seeding and thymocyte development by E2A-PBX1-transduced cells and a dramatic expansion of Gr-1^+^/CD11b^+^ myelomonocytic progenitors in the bone marrow and spleen (Fig 1). These findings are corroborated in our *in vitro* FLP model: the lymphoid potential of lin^-^ FLPs expressing E2A-PBX1 is blocked prior to the CLP stage (Fig 5), these FLPs are amenable to myeloid differentiation induced by GM-CSF, and they confer myeloid leukemia upon transplanted mice (Fig 4). We infer from our findings that the myeloid phenotype of the neoplasms that generally develop in mice transplanted with E2A-PBX1-expressing bone marrow is explained by a profound reduction of T- and B-lymphoid potential that is imposed early, prior to the CLP stage, coupled with retention of myeloid lineage potential. We speculate that the T-progenitor neoplasms that occasionally develop in mice transplanted with E2A-PBX1-transduced bone marrow cells may be explicable based on the relatively greater ability of E2A-PBX1-expressing progenitors to penetrate into the pool of committed T- relative to B-lymphoid progenitors (compare Fig 1A and 1C)[5].

Two of the genes that we demonstrate to be up-regulated in E2A-PBX1-expressing FLPs, *Mycn* and *Meis1* (Fig 6B), were identified recently among genes that were down-regulated in early myeloid progenitors from *Pbx1*-null mice, suggesting an overlap between genes regulated by wild-type Pbx1 and E2A-PBX1 in the myeloid context [45]. Meis1 is also a dimerization partner of wild-type Pbx1 [46]. *Meis1* expression has been associated with both increased self-renewal and increased SCF responsiveness [47;48]. Hoxa9 can block myeloid differentiation, immortalize myeloid progenitor cells and collaborate with Meis1 and E2A-PBX1 in the induction of murine AML [4;32;49;50]. We and other investigators have shown that *Meis1* and *Hoxa9* are expressed in HSCs but subsequently down-regulated during differentiation (S3 Table) [51;52]. In investigating a possible role for Hoxa9 in mediating the effects of E2A-PBX1, we observed that when lin^-^ FLPs are infected with a Hoxa9-expressing retrovirus, only cells that express Hoxa9 especially abundantly manifest blocked B-lymphopoiesis; Hoxa9-expressing cells were enriched in cells expressing CD11b relative to cells infected with an E2A-PBX1 virus (compare Figs 7C and 2A), suggesting that Hoxa9 is less effective than E2A-PBX1 in blocking myeloid differentiation. These observations suggest that factors in addition to Hoxa9 contribute to the differentiation block imposed by E2A-PBX1. Especially pertinent to this possibility are the findings of Wang and colleagues, who showed that lin^-^, bone marrow-derived progenitors subjected to retrovirus-enforced co-expression of *Hoxa9* and *Meis1* were more impaired with respect to B-lymphopoiesis and more potent in initiating acute myeloid leukemia than were cells transduced with *Hoxa9* alone, suggesting that Hoxa9 and Meis1 may cooperate to mediate the hematopoietic effects of E2A-PBX1 that we observed in our experiments [53].

In conclusion, our results suggest that the failure of mouse bone marrow transplantation experiments to model to E2A-PBX1-induced B-lymphoid neoplasia is attributable to the ability of E2A-PBX1 to block B-lymphoid commitment prior to the CLP stage while permitting retention of myeloid potential.

## Supporting Information

S1 Fig Growth curves are shown from from lin^-^ fetal liver cells infected with a retroviral vector expressing E2A-PBX1b (EP1b, dashed lines) or a control, empty vector (MIEV, solid lines). Results are shown for experiments lasting 14 (red lines) or 15 (blue lines) days after retroviral transduction.
(TIF)

S2 Fig Effects of the functional induction of E2A-PBX1 on the morphology of B-lymphoid progenitors. B-lymphoid 3PER12 cells stably expressing EPΔ623ER and cultured for 72 hours in the presence of estradiol (E2) appeared small and refractile compared to control cells treated with ethanol alone (vehicle), suggesting the occurrence of widespread apoptosis.
(TIF)

S1 Table Antibodies used for flow cytometry. (DOCX)

S2 Table Genes expressed differentially at least four-fold in E2A-PBX1-expressing fetal liver progenitors relative to control pro-B-cells. (XLSX)

S3 Table Expression patterns of genes expressed differentially downstream of E2A-PBX1 in primary lin- and myeloid cells. (XLSX)

S4 Table Probe ID numbers of genes in KEGG pathways overrepresented by genes up-regulated downstream of E2A-PBX1. (XLSX)

S5 Table Probe ID numbers of genes in KEGG pathways overrepresented by genes down-regulated downstream of E2A-PBX1. (XLSX)
