# Supplementary figures and images for: Retrovirus-Mediated Expression of E2A-PBX1 Blocks Lymphoid Fate but Permits Retention of Myeloid Potential in Early Hematopoietic Progenitors
Source: PLoS One. 2015 Jun 22;10(6):e0130495. doi: 10.1371/journal.pone.0130495 (PMC4476730; doi:10.1371/journal.pone.0130495)

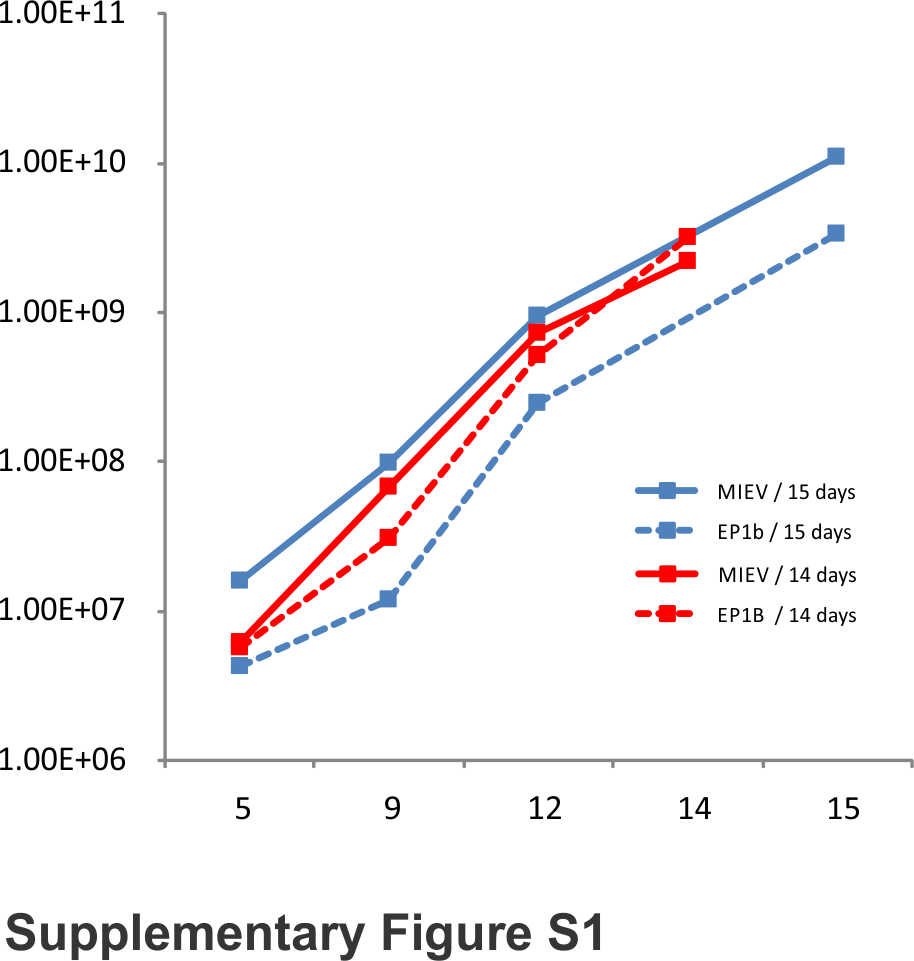

Supplement: S1 Fig — Results are shown for experiments lasting 14 (red lines) or 15 (blue lines) days after retroviral transduction. (TIF) [file pone.0130495.s001.tif]

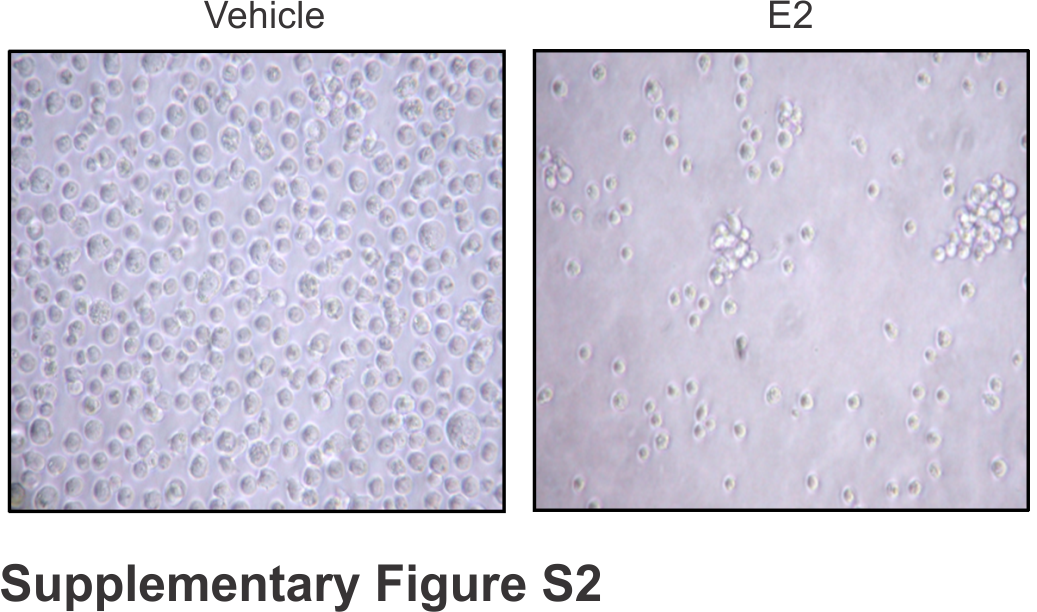

Supplement: S2 Fig — B-lymphoid 3PER12 cells stably expressing EPΔ623ER and cultured for 72 hours in the presence of estradiol (E2) appeared small and refractile compared to control cells treated with ethanol alone (vehicle), suggesting the occurrence of widespread apoptosis. (TIF) [file pone.0130495.s002.tif]
