# Supplementary material for: Retrovirus-Mediated Expression of E2A-PBX1 Blocks Lymphoid Fate but Permits Retention of Myeloid Potential in Early Hematopoietic Progenitors
Source: PLoS One. 2015 Jun 22;10(6):e0130495. doi: 10.1371/journal.pone.0130495 (PMC4476730; doi:10.1371/journal.pone.0130495)
Supplement: S1 Table — (DOCX) [file pone.0130495.s003.docx]

| **Supplementary Table S1**. Antibodies used for flow cytometry | | |
| --- | --- | --- |
|  |  |  |
| **Target** | **Clone** | **Fluorochrome** |
| **CD45R** | RA3-6B2 | PE/Cy5 |
| **CD19** | 6D5 | PE |
| **CD11b** | M1/70 | PE |
| **CD43** | S7 | PE |
| **CD49b** | DX5 | PE |
| **IL-7R** | A7R34 | PE |
| **Sca-1** | D7 | PE/Cy7 |
| **CD117** | 2B8 | PE |
| **F4/80** | BM8 | PE/Cy7 |
| **Gr-1** | RB6-8C5 | PE/Cy5 |
| **CD8** | 53-6.7 | PE/Cy5 |
| **CD4** | GK1.5 | PE |
| **CD3ε** | 145-2C11 | APC/Cy7 |
| **TER-119** | TER-119 | PE/Cy5 |
| **CD16/32** | 93 | N/A |
